# Supplementary material for: Plasmacytoid Dendritic Cell (pDC) Infiltration Correlate with Tumor Infiltrating Lymphocytes, Cancer Immunity, and Better Survival in Triple Negative Breast Cancer (TNBC) More Strongly than Conventional Dendritic Cell (cDC)
Source: Cancers (Basel). 2020 Nov 12;12(11):3342. doi: 10.3390/cancers12113342 (PMC7697894; doi:10.3390/cancers12113342)
Supplement: Supplementary file 1 [file cancers-12-03342-s001.pdf]

Supplementary Materials: Plasmacytoid Dendritic Cell (pDC) Infiltration Correlate with Tumor Infiltrating Lymphocytes, Cancer Immunity and Better Survival in Triple Negative Breast Cancer (TNBC) More Strongly than Conventional Dendritic Cell (cDC)

Masanori Oshi, Stephanie Newman, Yoshihisa Tokumaru, Li Yan, Ryusei Matsuyama, Pawel Kalinski, Itaru Endo and **Kazuaki Takabe**

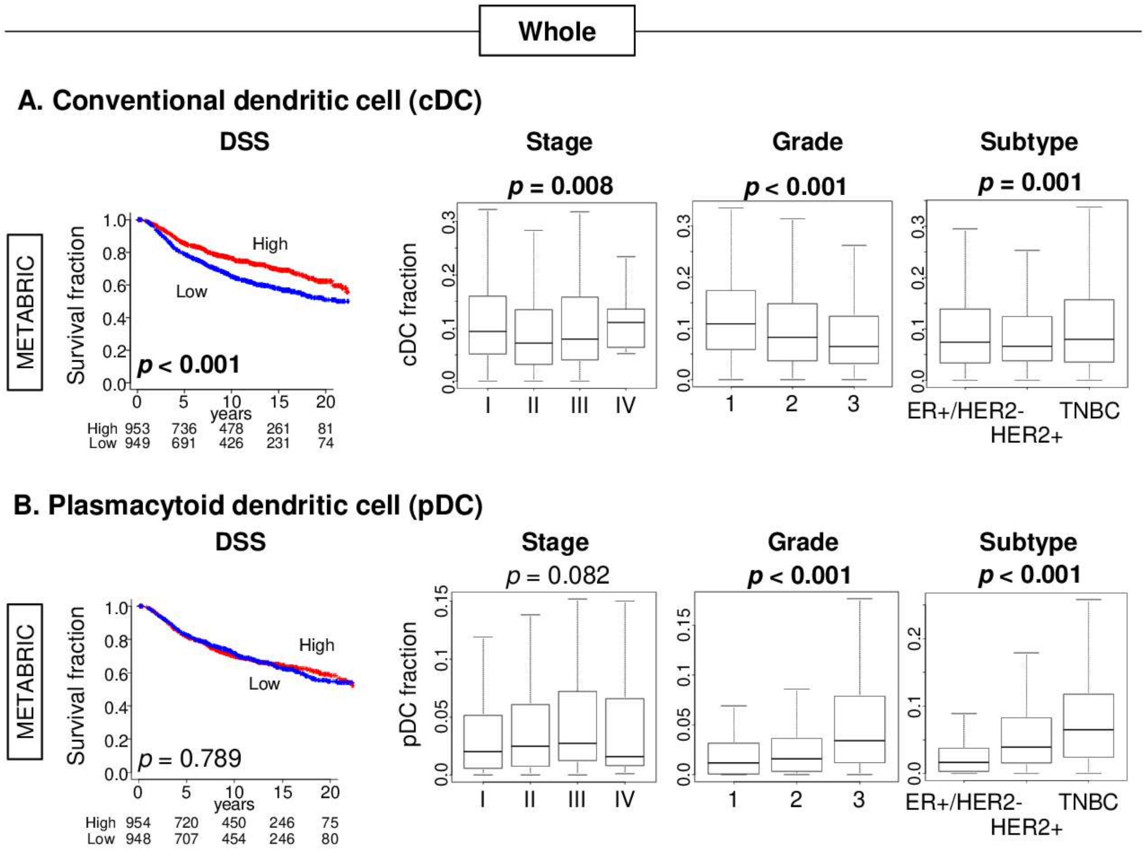

Figure S1. Association of conventional dendritic cell (cDC) and plasmacytoid dendritic cell (pDC) with clinical features in the METABRIC cohort. Kaplan-Meier survival (Disease-specific survival [DSS]) curves and boxplots of the AJCC stage, Nottingham pathological grade, and subtype with (**A**) cDC and (**B**) pDC. Kaplan-Meier curve show the DSS of cDC and pDC low (blue) and high (red) with *p*-value of log rank test. Median was used as cut-off to divide patients into low and high groups within cohort. Tukey type boxplots showed median and inter-quartile level values, and the One-way ANOVA test was used to calculate *p* values.

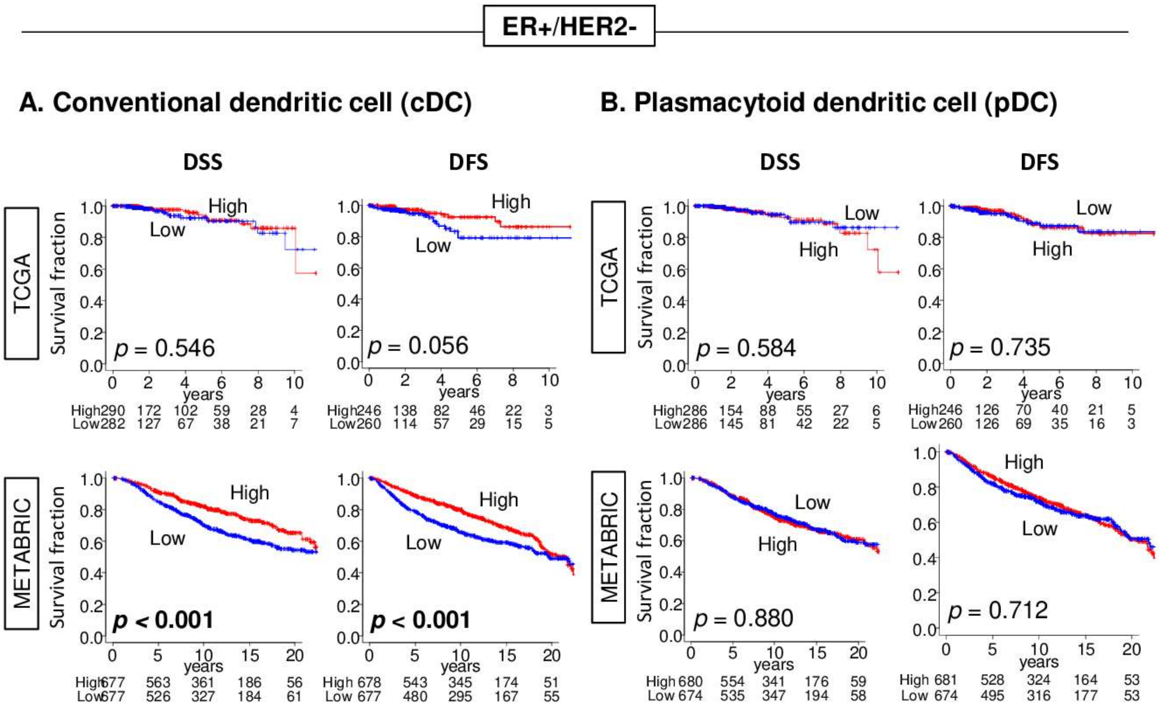

Figure S2. Association between cDC or pDC and survival of estrogen receptor (ER)-positive/human epidermal receptor 2 (HER2)-negative patients. Disease-free (DFS), disease-specific (DSS), and overall survival (OS) of (**A**) cDC and (**B**) pDC low (blue) and high (red) in ER-positive/HER2-negative cohort. Median was used as cut-off to divide into low and high groups within each cohort. Log rank test was used to compare between two groups with Kaplan-Meier survival curves and to calculate *p* value.

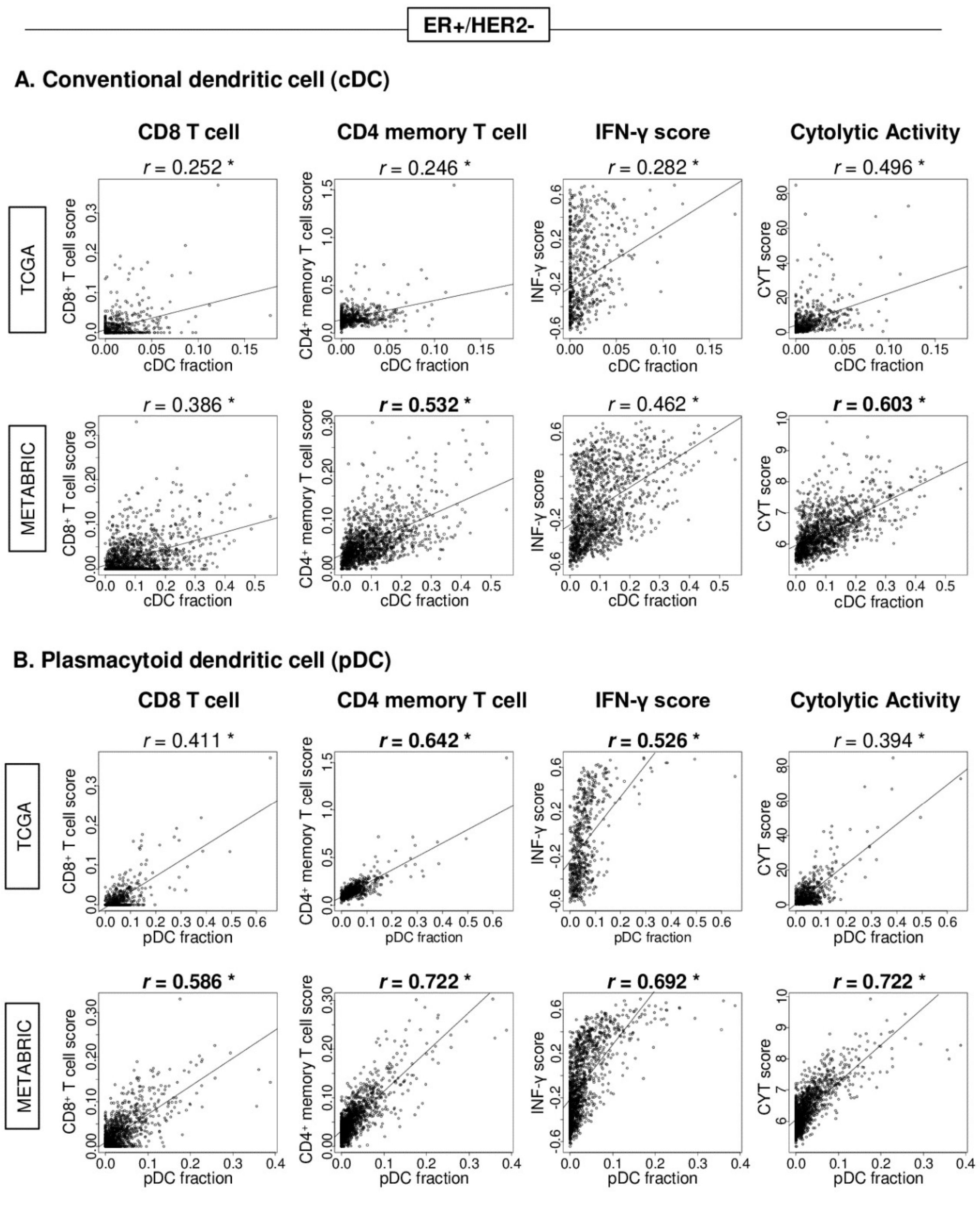

Figure S3. Correlation analysis of cDC or pDC in ER-positive/HER2-negative with $CD8^+$ T cell, $CD4^+$ memory T cell, IFN-γ pathway, and cytolytic activity score (CYT). Correlation plots of the (**A**) cDC or (**B**) pDC with $CD8^+$ T cell, $CD4^+$ memory T cell, IFN-γ pathway score and CYT. *p*-value was analyzed with spearman *r* correlation. * *p*-value < 0.01.

Table S1. Marker genes used to define conventional dendritic cell (cDC) and plasmacytoid dendritic cell (pDC) by xCell.

**cDC (38 genes)**		**pDC (38 genes)**	
Gene Name	Gene Description	Gene Name	Gene Description
ACTR3	actin related protein 3	APOC3	apolipoprotein C3
ALCAM	activated leukocyte cell adhesion molecule	CACNB1	calcium voltage-gated channel auxiliary subunit beta 1
ALDH1A2	aldehyde dehydrogenase 1 family member A2	CCR2	C-C chemokine receptor type 2
ALOX15	arachidonate 15-lipoxygenase	CD2AP	CD2 associated protein
ANXA1	annexin A1	CELA2A	chymotrypsin like elastase 2A
CCDC88A	coiled-coil domain containing 88A	CSHL1	Chorionic somatomammotropin hormone like 1
CCL13	C-C motif chemokine ligand 13	CUX2	cut like homeobox 2
CCL17	C-C motif chemokine ligand 17	CXCL13	C-X-C motif chemokine ligand 13
CCL23	C-C motif chemokine ligand 23	CXCR3	C-X-C motif chemokine receptor 3
CCL24	C-C motif chemokine ligand 24	DNASE1L3	deoxyribonuclease 1 like 3
CD163	CD163 molecule	FKBP2	FKBP prolyl isomerase 2
CD1A	CD1a molecule	FLT3	fms related tyrosine kinase 3
CD1B	CD1b molecule	FUT7	fucosyltransferase 7
CD1C	CD1c molecule	GZMB	granzyme B
CD1E	CD1e molecule	HIST1H2BB	Histone cluster 1 H2B family member b
CD209	CD209 molecule	HPD	4-hydroxyphenylpyruvate dioxygenase
CD80	CD80 molecule	IDH3A	isocitrate dehydrogenase 3 (NAD(+)) alpha
CD86	CD86 molecule	IL3RA	interleukin 3 receptor subunit alpha
CD93	CD93 molecule	KCNA5	potassium voltage-gated channel subfamily A member 5
CLEC10A	C-type lectin domain containing 10A	KCNK10	potassium two pore domain channel subfamily K member 10
CLEC4A	C-type lectin domain family 4 member A	KCTD5	potassium channel tetramerization domain containing 5
CRH	corticotropin releasing hormone	LILRB4	leukocyte immunoglobulin like receptor B4
DBI	diazepam binding inhibitor	LRRC36	Leucine rich repeat containing 36
DNASE1L3	deoxyribonuclease 1 like 3	MAPKAPK2	mitogen-activated protein kinase-activated protein kinase 2
FCER1A	Fc fragment of IgE receptor Ia	MYBPC1	myosin binding protein C, slow type
FGL2	fibrinogen like 2	P2RY14	purinergic receptor P2Y14
FLT3	fms related tyrosine kinase 3	PTCRA	pre T cell antigen receptor alpha
GFRA2	GDNF family receptor alpha 2	RPL3L	ribosomal protein L3 like
ITGAX	integrin subunit alpha X	RUNX2	runt related transcription factor 2
KCNK13	potassium two pore domain channel subfamily K member 13	SCT	secretin
PITPNA	phosphatidylinositol transfer protein alpha	SLC12A3	solute carrier family 12 member 3
RAB7A	RAB7A, member RAS oncogene family	SLITRK3	SLIT and NTRK like family member 3

RRP1B	ribosomal RNA processing 1B	SPCS1	signal peptidase complex subunit 1
S100A10	S100 calcium binding protein A10	SPIB	Spi-B transcription factor
SLAMF8	SLAM family member 8	TACR1	tachykinin receptor 1
SSR1	signal sequence receptor subunit 1	TLR7	toll like receptor 7
TCTN3	tectonic family member 3	TSPAN13	tetraspanin 13
WDFY3	WD repeat and FYVE domain containing 3	ZNF221	Zinc finger protein 221

© 2020 by the authors. Licensee MDPI, Basel, Switzerland. This article is an open access article distributed under the terms and conditions of the Creative Commons Attribution (CC BY) license (http://creativecommons.org/licenses/by/4.0/).
